# An Assessment of Wildlife Use by Northern Laos Nationals

**DOI:** 10.3390/ani10040685

**Published:** 2020-04-15

**Authors:** Elizabeth Oneita Davis, Jenny Anne Glikman

**Affiliations:** San Diego Zoo Institute for Conservation Research, 15600 San Pasqual Valley Rd, Escondido, CA 92026, USA; jaopy@hotmail.com

**Keywords:** wildlife trade, wildlife medicine, serow, bear bile, Laos

## Abstract

**Simple Summary:**

Although unsustainable wildlife consumption is a leading threat to biodiversity in Southeast Asia, there is still a notable lack of research around the issue, particularly into which animals may be “on the horizon” of impending conservation concern. Using semistructured interviews, we investigated the consumption of wildlife in northern Laos, with a focus on the use of wildlife for medicinal purposes. Bear bile was the most popular product, but serow bile was second in popularity and used for similar ailments. In light of these results, and considering the vulnerability of both bear and serow populations in the wild, greater concern needs to be taken to reduce demand for these products, before this demand becomes a significant conservation challenge.

**Abstract:**

Unsustainable wildlife trade is a well-publicized area of international concern in Laos. Historically rich in both ethnic and biological diversity, Laos has emerged in recent years as a nexus for cross-border trade in floral and faunal wildlife, including endangered and threatened species. However, there has been little sustained research into the scale and scope of consumption of wildlife by Laos nationals themselves. Here, we conducted 100 semistructured interviews to gain a snapshot of consumption of wildlife in northern Laos, where international and in some cases illegal wildlife trade is known to occur. We found that although bear bile for medicine was the most common product consumed, individuals also used a variety of other products, including animals considered endangered by the International Union for Conservation of Nature IUCN. The majority of animals we found consumed are classified as “Vulnerable” or “Least Threatened” by the IUCN; however, sufficient demand for a species can cause increased, rapid decline in the species’ population and significantly increase the challenge of conserving them. These results therefore illuminate where conservation priorities should shift towards, so that stable-yet-consumed species do not mirror the fate of highly trafficked animals.

## 1. Introduction

Unsustainable wildlife trade is a pressing conservation concern across the world and particularly in Southeast Asia, where accessibility to a rich array of wildlife facilitates widespread trade activity [1]. This use takes many forms, ranging from the “Asian songbird crisis,” where threatened songbird species are taken into captivity [2], to the well-publicized demand for rhino horn for medicine in Vietnam [3], to the consumption of bear bile for medicine in Cambodia [4], to many more examples of unsustainable demand and rapid decline. Yet, despite increasing global attention on unsustainable wildlife trade, many species and regions continue to be neglected in research and policy [5]. This is the case for many countries within Southeast Asia, perhaps due to adjacency to China and Vietnam, which are arguably the largest wildlife consumer markets [6,7] and thus the focus of much of the prevailing wildlife trade research. This is exemplified in the organizational structure of TRAFFIC, one of the largest wildlife trade-focused nongovernmental organizations (NGOs). TRAFFIC has dedicated offices in Vietnam and China, but has only a general “Southeast Asia” office, based in Kuala Lumpur, Malaysia [8]. Consequently, more remote areas of Malaysia such as Sarawak and Sabah, as well as other countries in Southeast Asia such as Cambodia and the Lao People’s Democratic Republic (hereafter “Laos”), can be neglected in wildlife trade research efforts, despite likely unsustainable consumption and known wildlife depletion in these countries [4,9,10,11]. Here, we focus on Laos, where wildlife trade is rampant across the Chinese border for the Chinese market [12,13,14] and a plethora of wildlife farms have been established to fulfill this demand [13,14], but where very little is known about the consumption habits of the country’s inhabitants.

The country of Laos is rich in biologic and ethnic diversity [15,16]. In Luang Prabang in particular, there are six “primary” ethnic groups: the Lao Loum, the Lue, the Hmong, the Khmu, the Iu Mien, and the Tai Dam. Other ethnic groups can be found in northern Laos, including the Rmeet [17], the Akha (e.g., [18]), the Tai-Cao [19], and many more. In our study, we sampled only Lao Loum, Lue, Hmong, and Khmu. The Lao Loum are the “dominant” ethnic group in Laos, and are descended from the Tai peoples who migrated from southern China in the high middle ages AD [20]. The Tai settled in the lowlands of Laos and Thailand, and are Theravada Buddhist. The Hmong migrated into Laos from China in the eighteenth century, and are generally mountain-dwelling and animist [21]. The Khmu are the “original peoples,” and share their language and heritage with the Khmer of Cambodia, although they are generally animist, compared to the Buddhist Khmer [22]. The Lue are also of the Tai peoples, but have retained autonomy from that ethnic group, with a distinct language and societal structure [23].

**Figure 1:**
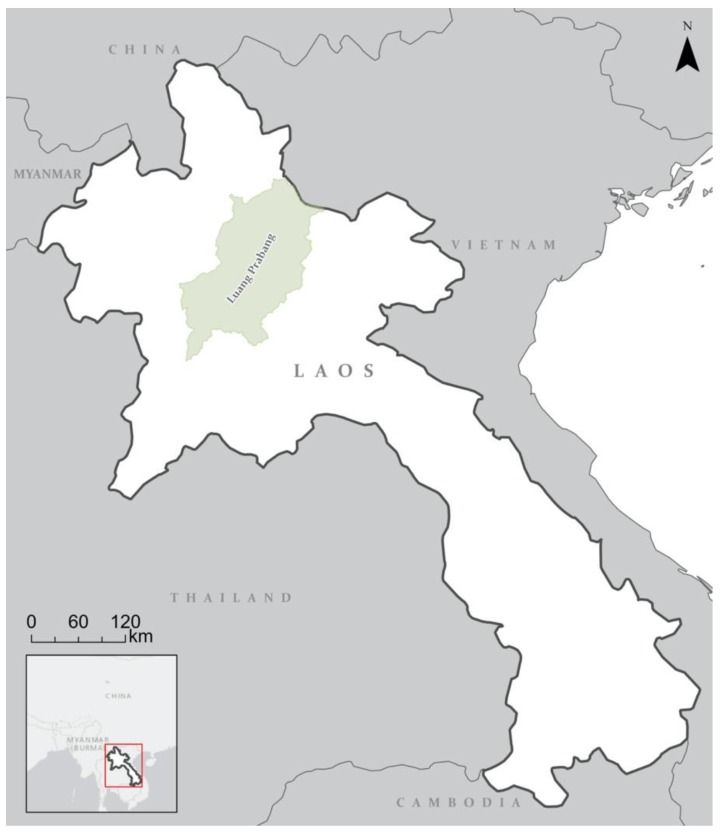
A map of Laos. Luang Prabang is indicated in the northeast of the country. As can be seen, Laos borders five countries: China, Myanmar, Vietnam, Cambodia, and Thailand (Map created by J. Stacey-Dawes).

The many ethnic groups of Laos have historically consumed the abundance of wildlife within the country, and for centuries this practice was sustainable. However, Laos’ position situated between what are now Thailand, Cambodia, Vietnam, Myanmar, and China (Figure 1), has sanctified Laos as a ready supply source for the recent, soaring demand of neighboring countries (e.g., [13,14,15,24]). This external demand from China and Vietnam in particular has undoubtedly caused the in-country extirpation of such iconic species as the Javan rhinoceros (*Rhinoceros sondaicus annamiticus*) [25] and, as recently as 2019, the tiger (*Panthera tigris*) [11]. The “porous borders” of Laos that wildlife flow through [26] do need greater enforcement and oversight from the Laos government to address the external threat from China. Concurrently, it is an undeniable truth that reducing consumer demand is imperative for preserving what species are left [27], particularly in Laos where wildlife consumption constitutes an “everyday activity” by Laos individuals [10]. Significant attention has been paid to the major consumer countries of China (e.g., [28]) and Vietnam (e.g., [3,29]), although several wildlife consumption assessments were conducted in Laos the early 2000s [10,30,31]. However, in the past decade little attention has been paid to consumption within Laos, although a study published last year in Khammouane Province (bordering Thailand and Vietnam) found a plethora of animals for sale in local wildlife markets, including the International Union for Conservation of Nature (IUCN)-designated Critically Endangered pangolin (*Manis* spp.) and other Vulnerable and Endangered animals such as sun bear (*Helarctos malayanus*) and Asian elephant (*Elephas maximus*), respectively [32]. It is now well-documented that sun bears and Asiatic black bears (*Ursus thibetnaus*) are currently being consumed in Laos [33,34,35]. More broadly, understanding the current consumptive landscape in Laos, and by extension the potential threats of demand to wildlife, is of particular necessity when considering recent assessments that have identified Laos as being in the “top 5%” of global areas that should be prioritized in conservation, based on the unsustainability and breadth of species being harvested [1]. 

Here, we present an updated view of wildlife consumption in northern Laos, an international wildlife trade hotspot [13,14,24]. Through semi-structured interviews and open-ended questions around wildlife, we explore present trends in consumption, and identify potential emerging trends in consumption. This is vitally important for understanding what species may be at risk of next suffering decline and eventual extirpation within Laos. Our results also provide a baseline of prevalence for consumption, that can be validated in future studies with large sample sizes.

## 2. Materials and Methods 

### 2.1. Study Site

This research was undertaken in villages surrounding Luang Prabang, a northern Laos town (Figure 1) that was the former home of the Laos royal family and a holy site for Theravada Buddhism [36]. 

Currently, Luang Prabang is a premier tourist destination in Laos for Western tourists, as well as Chinese nationals [33]. Some Chinese tourists are known to purchase wildlife products. As recently as 2017 ivory was openly sold at shops on the Luang Prabang high street [37], and a new market in Luang Prabang, built primarily for Chinese tourists, periodically has wildlife for sale (M. Hunt, personal communication). Separately from Chinese nationals, Laos nationals living in Luang Prabang are also known to consume at least two wildlife species: Asiatic black bears and sun bears [33]. 

The landscape around Luang Prabang is thick forest, with some small-scale farming of rice fields [38], and some larger Chinese initiatives growing crops such as watermelons (E. Davis, personal observation). Near Luang Prabang a biodiversity preserve has been established that connects the two wildlife sanctuaries of Tat Kuang Si Bear Rescue Centre, and the Luang Prabang Wildlife Sanctuary. This preserve has been given verbal support by the Laos Department of Forestry to receive targeted enforcement efforts; however, there has been little movement towards that point to date (M. Hunt, personal communication).

**Figure 2:**
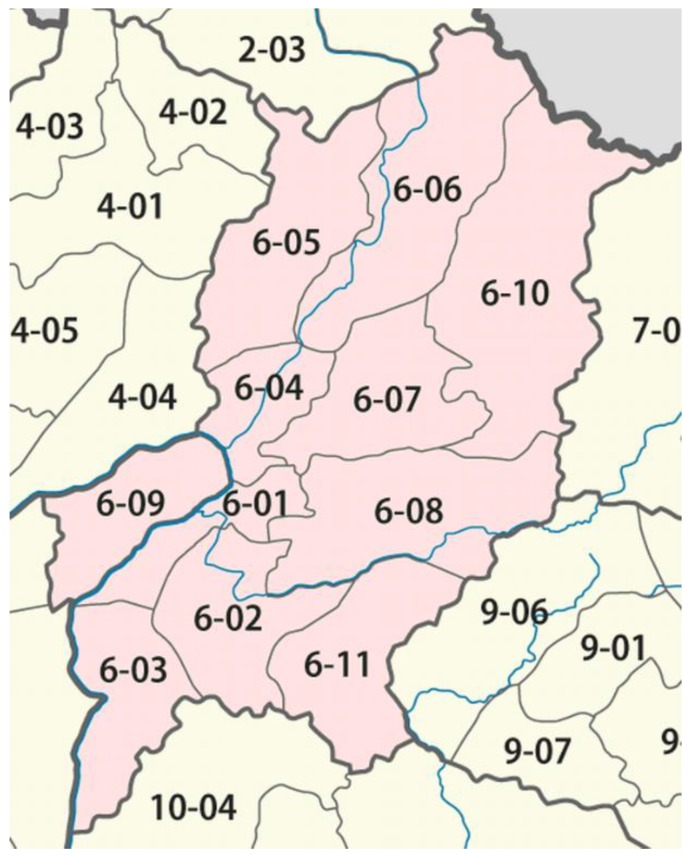
Map of the districts in Luang Prabang Province. Luang Prabang Town is in District 6-01 [39].

We conducted sampling in villages in Luang Prabang District (6-01) of Luang Prabang Province (Figure 2), Chompet District (6-09), Pak Ou District (6-04), and Phonxay District (6-08).

The villages exhibited a range of characteristics, from predominantly being Hmong, to lying adjacent to Chinese development projects, to being situated near one of many elephant sanctuaries in the area. The full table and list of characteristics of the villages is given in Table 1.

### 2.2. Survey Instrument

We performed semi-structured interviews using semirandom/convenience sampling. Individuals were approached, generally along the roadside and sometimes at their homes. We switched off between genders as we interviewed, and obtained a gender ratio of 51 females and 49 males. We performed 100 interviews in total. Interviews were conducted by the Lue interpreter in Lao (the “dominant” language) and in Lue (an ethnic minority language). The lead author guided all of the interviews, and recorded the responses throughout the interview.

The interview guide was adapted from a previous instrument used to perform interviews into use of bear products in Luang Prabang [34]. For this study, the instrument was adapted to incorporate questions that could act as a springboard for discussing multiple types of wildlife use. The instrument also encompassed a longer section on the medicinal systems (e.g., traditional, Western) used by the individuals sampled. The full instrument can be found in the Supplementary Materials (Guide S1). 

All interviews were anonymous and interviewees were asked for their verbal consent and told that they could stop the interview at any time. Permission to perform interviews was given by Miami University Research Compliance (Ref. #01334e), as well as the University of Bristol Faculty of Arts Ethics Committee. The Luang Prabang Tourism Office gave permission to perform interviews in Luang Prabang and the surrounding areas.

## 3. Results

### 3.1. Demographics

The average age of the sample was 43 years, with the median 44.5. The age range was 20–72. Lao Loum were found to be the most predominant ethnic group represented (81%), followed by Hmong (10%), Khmu (6%), and Lue (3%). The most predominant occupation was as a food or drink seller, generally at a roadside stall (*n* = 27). This occupation was predominantly composed of women (4:23). The second most common occupation was none, specifically “housewife/husband and/or retired” (*n* = 11, 4:15). The third most common occupation was as a farmer (*n* = 11, 8:3). The full list of occupations can be found in the Supplementary Materials.

Most of the individuals interviewed had spent the majority of their life in either Luang Prabang District (64%; 6-01 in Figure 2) or in Chompet District, across the Mekong River from Luang Prabang town (17%; 6-09).

The predominant religion in the sample was Buddhism (85%). Animism was practiced by another 12% of the sample, followed by 2% who practiced both Buddhism and animism together. One individual was Christian.

Less than a fifth of the sample owned a car for activities (19%). Cars are status symbols in Asia [40], indicating that greater wealth is needed to purchase a car. Additionally, the occupations stated above indicate that the sample was predominantly middle status in northern Laos, being neither overly low status or high status.

### 3.2. Use of Wildlife

A diversity of wild animal products was stated as being consumed by the interviewees (Table 2). Other products not used by the respondents, but stated to have been used by other known individuals were elephant (*Elephas maximus*) skin, deer (*Cervidae* spp.) bile, chicken wattle, “wild chicken,” a “big bat,” bone of monkey hand, and a “special type of fish.”

Bear bile is by far the most popular wildlife product, but serow is also a relatively widely consumed product (Table 2). According to the interviewees, it is used for much the same rationale as bear bile/gallbladder, which is generally used as a topical application or consumable tonic to treat bruising, particularly after motorbike accidents, or mixed with whiskey and drunk to “reduce fatigue.” Beyond this sample, individuals widely discussed serow, and particularly serow fat, as having been used by other members of their close social network, such as husbands, sons, brothers, and grandmothers. Individuals stated that themselves and/or their social network used it as a balm on bruises, sprained ankles, and in certain cases, mixed with whiskey for addressing fatigue.

This was also true of “wild buffalo,” probably gaur, bile. One 28-year-old male Lao Loum respondent stated that gaur bile is highly effective after motorbike accidents, and gave the following anecdote as his support for this belief:

My brother fell off his motorbike. Doctors said that he would die. But a Hmong man gave us wild buffalo bile to give to him, and he lived. The man gave it to us at the hospital.

This anecdote represents an extraordinary altruistic act between strangers, considering the price that gaur bile can be (seen Table 3). It also supports the widely held beliefs of the Tai groups of Lao Loum and Lue that the Hmong have a greater understanding of “how to use” wildlife products, particularly for medicine [37]. However, this is challenged by one 35-year-old Hmong male’s view:

Old Hmong people say that bear bile cures fatigue. Mix it with warm water. I don’t believe it though because I have never used.

One 49-year-old Hmong farmer had used rhino horn in the past, and it is entirely possible that he and/or his family sourced the rhino directly from Laos; the last population of the Javan rhinoceros is believed to have been extirpated from Laos sometime in the 1990s [25].

Another dimension of wildlife consumption in northern Laos is the prices quoted for the products (Table 3). 

Prices for the other wild animal products are generally much lower than bear bile/gallbladder, except for the significant outlier of buffalo (probably gaur) bile, at well over 2000 USD (Table 3). This is likely a reflection of gaur’s rarity and the transport involved in obtaining the product. This is supported by the statement of the individual who had consumed it, who said that his friend had purchased the bile at the Thai–Laos border, which likely necessitated costs in terms of getting the product to that area. In the 1990s, gaur were still found in Laos and therefore it would have been relatively accessible for individuals to consume those products [26]; however, it is possible that they have now been extirpated, which could be why the individual in this sample had to source it from Thailand, if not beyond.

### 3.3. Wildlife as Medicine

The interviewees were asked: “Are wildlife products part of traditional medicine?”, and 48% of the individuals stated that they believed that wildlife products are part of traditional medicine. Usually this knowledge came from having used wildlife products themselves to heal some ailment, or knowing of traditional remedies that used wildlife products. As noted above, a plethora of wildlife products were cited, including multiple animals’ skin, wild pig gonads, “wild goat” (i.e., serow), and “wild buffalo” (possibly gaur). The most commonly cited product was bear bile.

However, the majority of the sample did not believe that wild products were part of traditional medicine (52%, *n* = 52). Reasons for this included perceived negative health benefits of wild animals, i.e., that consuming them would give high blood pressure. Other reasons given were a lack of knowledge. For example, a 55-year-old male, Hmong farmer said:

I think it can be traditional medicine, but there is no wildlife around now and/or people are not using it, so I’m not sure how to answer.

This indicates that rooted knowledge in traditional medicine (TM) is not high around Luang Prabang. Older individuals would theoretically have been exposed to TM more than younger individuals, yet there was a general lack of consensus as to what constituted TM among the sample, and whether wildlife products constituted part of the TM system. There was also a lack of strong belief in TM’s efficacy in general, with only 41% of the interviewees stating that they believed it was “good.” However, only 6% of the interviewees declared the reverse and that they did not believe it and did not think it was effective. The majority of the interviewees were somewhere in the middle, and unwilling to give a strong opinion either way. This was attributed generally to a lack of knowledge, with respondents stating “don’t know about it,” and one 35-year-old Lue woman stating:

It’s good if you know how to use it, but now so few people know how to make TM mixtures.

One Lao Loum woman interviewed said that going to the biomedicine clinic cost approximately 0.75 USD, so there was no cost barrier to receiving “Western” healthcare. Additionally, the roads have greatly improved in the past 10–20 years, so getting to and from these healthcare centers is significantly easier. Individuals in Ban Xieng Lom, a village about thirty min. due east of Luang Prabang along the Nam Khan, stated that once the road from their village to Luang Prabang town was paved and it was consequently much easier to get to the Luang Prabang hospital, use of TM drastically decreased. 

Individuals who used wildlife for medicine stated that they used it due to recommendations from family or friends. One elderly Lao Loum woman told us that when her husband was alive he wanted them to take bear bile wine for “health.” When he died, she stopped using it. Three individuals interviewed stated specifically that although they drank bear bile wine to cure fatigue, it was drunk socially with a group of friends, and they did not drink it in other settings. This context of being given the product by close members of the social group was also true of serow fat, which was also stated to have been given by friends and family. No one interviewed had sought out serow fat; rather, they were given it by someone in their social network, and in at least one case they were specifically given it as a gift. The individual who used tiger bone was not clear on how he had obtained it (possibly due to greater sensitivity around the use of tiger compared to other wildlife products), but this too could possibly have been a gift.

## 4. Discussion

Laos is one of the world’s premier wildlife trade hotspots (e.g., [1,11,13,14,24,32,37]), with the narrative around Laos in the past decade that of a “source” site exploited by Chinese consumers [14,24] and Vietnamese consumers [6,41]. Although it is undeniable that this outside demand is a significant concern, research into the patterns of consumption by Laos nationals themselves have largely been neglected. One general finding from our results, with significant implications for conservation, is that wildlife is widely consumed around Luang Prabang. Nearly a third of the sample had consumed bear bile/gallbladder, followed by different products from serow. It is well-known that bear bile/gallbladder is widely consumed in this study area in northern Laos [33,34,35]; however, this study has shown that use may be significantly higher than previously recorded [33]. This study is also the first to provide a comprehensive snapshot of other wildlife use, particularly serow. Mainland serow (*Capricornis sumatraensis*, previously specified as “Chinese serow” [42]) is a medium-sized ungulate found in the mountainous regions of northern Laos, and will shortly be uplisted from “Near Threatened” to “Vulnerable” (S. Lovari, personal communication). In a previous conservation status assessment of serow, a significant threat was noted of serow products being “highly prized” for many uses, including for medicinal purposes [43]. However, this threat was primarily identified as being in “the northern parts of its range,” which is northern China [43]. Our research here provides supporting evidence that serow is also being hunted in the southern parts of its range, for the same reasons. Apart from Laos, serow has also been documented as traded for medicine in Myanmar [44,45]. To our knowledge, there have been no clinical studies into serow’s actual medicinal efficacy. However, the ubiquity of its use may indicate that it does have efficacy in treating some ailments. In this respect it appears to follow use of bear bile, which is historically and into the present widespread across East and Southeast Asia [29,34], possibly as a result of real efficacy in treating certain ailments [46].

There is less need for traditional medicine in this area of northern Laos, as Western medicinal structures including clinics and hospitals have been integrated into villages across the region, and are very cheap for Laos individuals. Indeed, our results show that wildlife is not strongly thought of around Luang Prabang as “traditional medicine.” In addition, from these results and previous research in the area [37], it seems probable that the younger generation of all ethnic groups are increasingly less likely to use wildlife products, which could be due to the widespread decline in the available supply of wildlife in Laos (despite a lack of broad assessments into wildlife population numbers), and/or a genuine decline in desire to use wildlife. As previously discussed, this area of Laos has undergone significant changes in the availability and accessibility of healthcare in the last few decades, and therefore there is less necessity to rely on traditional medicine alternatives.

Rather than acting as strictly utilitarian and medicinal, it is possible that the use of wildlife for medicinal purposes exists in an “other” space, where use is determined by peers and family [37] and by the process of gift-giving. This is true even of those groups, like the Hmong, who are documented as having a long association with wildlife and a diverse traditional medicine system that includes consumption of wildlife, including bear bile [47]. Bourdieu [48] writes that gift-giving is essentially overlaid on the “raw” economic consumptive framework, and equally “essential” to society. This may be especially true in collectivist, i.e., group-oriented societies, which are argued to be predominant in East and Southeast Asia (e.g., [49]). Giving gifts maintains social relations, drives social relationships, and can increase one’s social capital and/or social approval and by extension prestige [50]. This is exemplified by the giving of bear bile as a gift in Vietnam [29]; the gift of medicine holds powerful connotations of care and compassion [51], and with successful treatment of the receiver’s ailment comes the obligation to return such care and compassion to the “original” giver. As shown here, this could easily be in the form of reciprocal giving of bear bile or another analogous wildlife medicinal product such as serow, as an “equal” exchange. 

Another important facet of wildlife trade is authenticity of the products. The low prices quoted for some of the products prompted the interpreter to suggest that they were fakes. “Fakes” generally are domestic animal products sold as the wild product. For example, fake bear bile is often pig bile (B. Crudge, personal communication). It is also worth noting here that of the animals consumed, two taxa (tigers and bears) are known to be kept at farms in the nearby area. Although it is unknown whether the tiger bone used was believed to have been obtained from a farm or the wild (or indeed whether it was tiger at all), the bear bile/gallbladder consumed was believed to be mostly wild-sourced. Only 2% of the stated bear bile users believed that they had consumed farmed bear bile/gallbladder, and both individuals stated that it had come from outside the Luang Prabang area: one was from China, and the other was from the Vientiane area. 

A potential confounding factor is the interview guide itself, which was skewed towards discussion of bear products; however, because wildlife and traditional medicine were discussed at the beginning of the questionnaire, individuals who had used wildlife for medicine would state all products they had used in the “Wildlife Use” section of the questionnaire, regardless of the guiding questions about bear product use. However, the questionnaire’s emphasis on bear bile meant that less discussion was given to other wildlife products’ use for treating certain ailments, and so a useful opportunity for future research would be understanding the specific ailments that serow is used for, and whether serow products are ever used to treat severe ailments. 

## 5. Conclusions

Here, we show that the use of wildlife products continues apace in the Luang Prabang area, despite the efforts of the Laos government to address wildlife use according to repeated warnings from Convention on International Trade in Endangered Species of Wild Fauna and Flora (CITES) [52] and recognition of the continued decline of wildlife population in the country [1]. Our results of present and prevalent demand for wildlife in northern Laos indicates both that enforcement efforts are not working and that the Laos government’s goals of reducing wildlife trade may be challenging to achieve.

Our results also indicate the importance of identifying emerging trends in wildlife consumption, which can inform efforts to halt population declines before they become full blown crises. We have shown that serow is the second most consumed wildlife product by Laos nationals around Luang Prabang. The current harvest of serow in Laos may not have a significant impact on populations at present; however, it is also possible that the current harvest is unsustainable, and could become exacerbated if a significant number of bear medicine consumers turn to using cheaper serow products instead. If serow consumption follows the trend of bear product consumption (e.g. [53]), the population may suffer a sudden, serious, and rapid decline in the next decade, which will hinder future effective conservation efforts. Indeed, the clearly unsustainable nature of wildlife trade and consumption broadly in Laos necessitates solutions beyond the status quo of enforcement. A recent review emphasized “adaptive” wildlife trade countermeasures [54], and we argue that the research presented here represents a critical first step towards a forward thinking, progressive conservation management solution in northern Laos that identifies and incorporates this emerging threat to wildlife such as serow, who may be excluded from management plans directed at conserving more “charismatic” fauna. 

We also note that the social motivations that drive wildlife trade will be challenging for conservation organizations to address when working to reduce demand for wildlife in northern Laos (and possibly throughout the country). Shifting these societal networks of exchange will necessitate thoughtful interventions that integrate these processes, as well as these identified key actors of, for example, givers and receivers. Future efforts should build on these findings and incorporate them into well-defined Theories of Change (ToCs) that identify and acknowledge the specific consumer profiles for each product [55], which can in turn be used to guide behavior change campaigns that employ conservation marketing techniques to give wildlife users positive and desirable alternatives [56].

## Figures and Tables

**Table 1:** Villages where the interviews (*n* = 100) were conducted.

Village	Number of Interviews	Defining Features
Ban Khoum Khoung	16	A large village that encompasses a broad area about 10 min. away from Ban Phan Luang. It is predominantly Lao Loum. Someone was selling tasters of bear bile (probably authentic, based on the price) in this village not very long ago.
Ban Xiengméne	16	The village just across the Mekong from Luang Prabang. There are regular ferries across and it is billed as a “heritage village” with regular farlang (Western foreigner) bicycle tours. It is also apparently reasonably popular for farlang who want to see Wat Chompet, and as a transit point for Thai and Chinese tourists going into Chompet District, some for the waterfall and zipline on that side.
Ban Xieng Lom	10	About thirty min. due east of Luang Prabang along the Nam Khan, along a paved road that has become torn to bits by the amount of Chinese construction going on around the area. Near Ban Xieng Lom is the Elephant Village, as well as the Spirit Resort and the Zen Namkhan Resort.
Ban Koy	10	About a 5 min. drive from the center of Luang Prabang. Most of the houses are big and people seem fairly prosperous compared to many other villages.
Ban Kouk Va	6	A Hmong village close to Souphanovoung University (approx. 20 min. from Luang Prabang town proper).
Ban Pa O	6	Along the Mekong, about thirty min. north of Luang Prabang town, past the Chinese railroad project and the accompanying Chinese-run warehouses/factories.
Ban Pakseung	6	About ten min. past Ban Pa O. An idyllic little town along the Mekong that must see some tourists because jet ski rides are offered. These appear to be predominantly marketed to Chinese tourists.
Ban Phaleuk Cheurn	6	Another Hmong village, near Souphanovoung University, approx. 20 min. from Luang Prabang.
Luang Prabang	4	The main town.
Ban Non Ka	4	One of the villages that comprise Luang Prabang town.
Ban Xiengkao	3	A somewhat isolated village south of Luang Prabang town, off a road before the road leading to Tat Kuang Si, further into Luang Prabang district.
Ban Noon Savaat	3	A village along the road leading to Ban Xieng Lom (due east of Luang Prabang). It is right in the center of substantial Chinese construction projects and industrial factories/warehouses. It is about 20 min. from LP.
Route 13	3	Two interviews conducted on Route 13 in the outskirts of Luang Prabang town and one interview conducted twenty to thirty min. up Route 13, towards Pa O town.
Ban Nouosai	2	One of the villages in the Luang Prabang town area.
Ban Sen Souk	2	Another village in the Pa O area
Ban Had No	2	A village along the main road heading west, in Chompet District. The villages along this road have very low density of people compared to villages in Luang Prabang District.
Ban Kum On	1	Near Luang Prabang and on a road leading to Tat Kuang Si.
Ban Sak Alou	1	Same as above.

**Table 2:** Types of animal products consumed by the interviewees (in %), along with the conservation status of the animal.

Animal Product	Percent of the Sample Who Have Used (%)	Conservation Status of the Species (with Assessment Year)
Bear bile/gallbladder (sun bear: *Helarctos malayanus* or Asiatic black bear: *Ursus thibetanus*)	26	Vulnerable (2016 and 2017)
Serow (*Capricornis sumatraensis)* fat	7	Not Threatened (2008) *
Wild meat (deer, frog (*Ranoidea* spp.), snake (*Serpentes* spp.), bird (*Aves* spp.), pig (*Sus* spp.), serow)	6	*Unknown which species but possibly threatened*
Wild buffalo bile (likely gaur (*Bos gaurus*))	3	Vulnerable (2016)
Wild pig gonads	2	Least Concern (2019)
Rhino horn (likely Javan: *Rhinoceros sondaicus*)	1	Critically Endangered (2008)
Deer bone	1	*Unknown which species but possibly threatened*
Serow bile	1	Not Threatened (2008) *
Serow bone	1	Not Threatened (2008) *
Snake bile	1	*Unknown which species but possibly threatened*
Wild buffalo (likely gaur) urine	1	Vulnerable (2016)
Water buffalo (*Bubalus bubalis)* bile	1	Domesticated
Wild pig tail	1	Least Concern (2019)
Wild pig urine	1	Least Concern (2019)
Tiger (*Panthera tigris*) bone	1	Endangered (2015)
Dog face	1	Domesticated
Porcupine (likely brush-tailed porcupine (*Atherurus macrourus*))	1	Least Concern (2020)
“Big squirrel” (possibly either Giant squirrel (*Ratufa bicolor*) or Giant flying squirrel (*Petaurista elegans* or *Biswamoyopterus laoensis*))	1	*R. bicolor*: Near Threatened (2016) *P. elegans:* Least Concern (2016) *B. laoensis:* Data Deficient (2017)

* Mainlad serow will be updated to “Vulnerable” in early 2020 (S. Lovari, personal communication).

**Table 3:** Average prices of wild animal products given by the sample (total *n* = 100).

Product	Price (USD)
Buffalo bile	2294
Bear bile (little bit)	70
Serow fat (little bit)	11
Serow meat	9
Wild pig meat	6
Wild pig tail	6
Wild pig urine	6
Serow gallbladder sliver	3

## Supplementary Materials

The following are available online at https://www.mdpi.com/2076-2615/10/4/685/s1: Guide S1: Interview Guide Luang Prabang.
