# Supplementary material for: An Assessment of Wildlife Use by Northern Laos Nationals
Source: Animals (Basel). 2020 Apr 15;10(4):685. doi: 10.3390/ani10040685 (PMC7222730; doi:10.3390/ani10040685)
Supplement: Supplementary file 1 [file animals-10-00685-s001.pdf]

Interview Guide Luang Prabang

Hello, as part of a research project for San Diego Zoo Global we are interested in gaining an understanding of people's relationships with wildlife. Anything that you tell me will only be used for the purpose of this research project and will be kept private and confidential. The interview does not request information that would explicitly identify you. In addition, results of the research will only be presented publicly as aggregate summaries. If you have questions or concerns about the rights of research subjects, you may contact our reviewing body: the Research Ethics and Integrity Office at Miami University at (513) 529-3600 or humansubjects@miamioh.edu.
Completing the survey should take about 30 minutes. *Participation is voluntary and you may withdraw at any time, without any consequences.* Are you happy to complete an interview with us?

Interviewee agreed to participate (tick if YES)	

Demographics

1. Age [make sure over 18] _________
2. Gender ________
3. Ethnic group ________
4. Occupation ___________
5. Were you born in this village? ____________
6. [If NO] Which village were you born in? _______________________ [also write province and district]
7. [If NO] How long have you lived in this village?_______________________
8. Where have you lived most of your life? ____________________
9. Do you own a car?

Health Care

10. As far you know, do people practice traditional medicine in this district?

11. If you were sick, what type(s) of healthcare would you use? When would you use TM (e.g before *farlang*? After?)?
12. Why do you use that type of healthcare?
13. In what contexts are different healthcare systems used? (e.g., disease/ accessibility/ cost/ tradition) ?
14. What do you think of when I say traditional medicine?
15. Do you think the proportion of people in Laos using traditional medicine has changed over the last 10 years?
16. [If YES] How and why do you think that is?
17. Do you think that wildlife products are part of traditional medicine? Why?
18. Do you know about traditional Chinese medicine?
19. [if YES] Do you think it differs to traditional medicine in Laos? How?
20. Do you think the proportion of people in Laos using traditional Chinese medicine has changed over the last 10 years?
21. [If YES] How and why do you think that is?
22. Do you think that wildlife products are part of traditional Chinese medicine? Why?
23. In the past twelve months did you use Western medicine?
24. In the past twelve months did you use traditional medicine?
25. Does your village have a shaman?
26. [If YES] Can you briefly explain what duties they tend to perform?
27. [If YES] Do you visit the shaman?
28. What religion(s) do you practice? (animism, Buddhism, etc) ______________

Wildlife use

1. Which of these medical behaviors have you done/medical ailments have you had *this year*? [refer to the spreadsheet]

2. Have you heard of any bear parts being used by someone?

3. If so, which ones and for what medicinal conditions?

4. Do you believe that bear products are TM?

5. Which medicinal bear product would you buy, if any?

6. How are the products you mentioned used? *(E.g. bear gallbladder mixed with forest dew?)*

7. How do you think people who use bear parts are perceived in your culture?

8. Have you ever used bear products? Who suggested them to you?

9. *[If they mention bear gallbladder/ bile]* Given the choice, would you prefer wild or farmed bear bile?

10. [*if they only mention medicinal bear products*] Do you know of bear products being used for non-medicinal purposes?

11. [if YES] Have you used these products? Who suggested them to you?

12. Would you consider buying a non-medicinal bear product?

13. [IF YES] which product would you consider buying?

14. Can you think of a reason why you would not use bear products?

15. Were you or someone in your family pregnant or have a baby in the last 12 months?

16. [if YES] Which medicines did you/she use during pregnancy or after childbirth?

17. [if YES] Did you/she use bear bile/gallbladder? If so, why? Who offered it to you?

18. How many members of your closest family and friends do you know <u>for certain</u> have used/consumed bear bile or gall bladder for medicinal purposes in the last six months?

Answer:__________ *(if 0 or 1, write the number and skip to 45. If >1 go to 44).*

Note: Write the initials of every individual on separate pieces of paper. If the interviewee knows 100 people that have used bear bile or gall bladder for medicinal purposes in the last six months then you will need 100 pieces of paper. Then ask the interviewee to select one piece of paper to identify the nominated friend. The pieces of paper will be disposed of.

19. Other than you, how many other people do you believe know that the nominated friend has used bear parts or products for medicine or other purposes?

Answer:__________ .

20. Have you used other wild animal products?

21. Can you tell me the price of the products you mentioned?

22. Finally, who are the most influential or most important people in your village? *(Encourage them to list as many as possible, not just one).*

Interviewer comments:

Interview notes (e.g. context, reliability):
